# Dr. Ottolengui Objects

**Published:** 1904-12

**Authors:** 


### DR. OTTOLENGUI OBJECTS.

In a private letter to the editor, Dr. Ottolengui states that the editorial in the October number of the International Dental Journal contains an erroneous statement in regard to the nominations for President of the Fourth International Dental Congress, held at St. Louis, August 29 to September 3. His statement is as follows:

"As I was personally associated with the party who advocated the election of Dr. Johnson, I beg leave respectfully to take exception to the way you have reported the matter in your editorial in the October number of the International Dental Journal. You say that the nominations for President were Dr. H. J. Burkhart and Dr. C. N. Johnson; also that the vote for both officers was taken by each member standing until the vote was counted. Will you pardon my reminding you that as a matter of fact Dr. Johnson was never placed in nomination at all? indeed, by the most arbitrary sort of ruling we were not permitted to introduce his name."

It is true that the ticket headed by Dr. Johnson for President was not nominated in the usual formal manner, and the editorial statement was thus far in error, but the difference between a formal nomination and a general open understanding that the ticket was prepared to be voted for at the proper moment is about the difference between tweedledum and tweedledee. Dr. Ottolengui was not permitted the floor by Mr. Rogers, the temporary President. The writer regarded this then, and still regards it, as the most arbitrary decision it has ever been his experience to meet with, coming from a President claiming any knowledge of parliamentary law, and under other circumstances should have been very properly resisted. While this was true, and the nomination was not permitted to be made properly, there was not a person familiar with the English language present who was not aware of the nominations to be made, and it was equally well understood that had Dr. Burkhart failed of an election, Dr. Johnson's name would have been presented to the Congress. The part of the editorial in question, in which it was stated, "The vote for both officers was taken by each member standing," had allusion to the offices of President and Secretary, and not to the two nominations for President.

That the writer is correct in stating that it was universally understood that Dr. Johnson was to be placed in nomination,—indeed, was practically nominated,—the following quotation from a St. Louis daily paper is given in evidence. After giving a very true and detailed account of the difficulty that led up to this contest, the report proceeds:

"The opposition retaliated yesterday by putting an independent ticket in the field. For President their choice is Dr. C. N. Johnson, of Chicago, editor of the *Dental Review*. Dr. Kirk became their candidate for Secretary. The issue has been clearly drawn, and was the absorbing undercurrent topic at the dentists' meetings yesterday. Some of the members declare that they will not be drawn into the wrangle, and will not take sides, but all are watching the outcome expectantly. The foreign delegates are standing aside and permitting the Americans to have it out. When approached on the matter they give a nod of the head and show an air of amused interest."

The fact that Dr. Johnson followed the usual custom of moving the unanimous election of Dr. Burkhart was sufficient proof, if any were needed, that he, at least, felt he was practically in nomination.

Space is given somewhat reluctantly to this somewhat extended explanation, but it is deemed due to the parties interested that the matter should be corrected.

The writer at the time had but a very mild interest in this contention for office. From first to last it was an undignified exhibition of a morbid ambition for office, that created a bad impression upon all reasonable thinking persons present. The writer was personally favorable to both nominees. He regarded either as eminently qualified for the responsible position, but his preference had been in the direction of an international character who fully represented both America and Europe. Such a nomination, had it been made, would have given character and dignity to the Congress. This view, however, received no consideration, and the elected President was placed in the unenviable position of being the head of a great congress without an address suitable for the occasion. Had the appointment been made months previously, this could have been avoided. The matter is now, however, past history, to be read and pondered over for the benefit of future dental congresses.

